# Sacral chordoma with incidental rectal adenocarcinoma: a case report

**DOI:** 10.1186/s13256-021-02728-2

**Published:** 2021-04-12

**Authors:** Jaffar Alshahri, Mohammed Alshehri, Aminah Alnafesa, Mohammed Widinly, Tariq Alzaid, Saleh Alsulaimani, Alaa Abduljabbar

**Affiliations:** 1 King Faisal Specialist Hospital and Research Center, Riyadh, Saudi Arabia; 2 King Saud Medical City, Riyadh, Saudi Arabia; 3 Security Forces Hospital, Makkah, Saudi Arabia

**Keywords:** Rectal cancer, Sacral chordoma, Sacrectomy, Case report

## Abstract

**Background:**

We report a unique case of synchronous sacrococcygeal chordoma in association with rectal invasive adenocarcinoma. Retrorectal tumors are a rare disease caused by a variety of pathologies. To our knowledge, no prior cases of such a coincidental finding of both cancers have been reported in the literature.

**Case presentation:**

This is the case of a 74-year-old white middle eastern man, with known hypertension under treatment, who presented with complaints of progressive lower back pain associated with urinary incontinence over the past 12 months. Magnetic resonance imaging (MRI) of the pelvis showed a large midline, well-defined, oval-shaped lesion replacing the sacrococcygeal portion of the spine, with extension to the presacral region. Computed tomography (CT)-guided Tru-Cut biopsy revealed features suggestive of chordoma. At surgery, we performed excision of the entire mass en bloc, sacrectomy with rectus abdominis myocutaneous flap reconstruction and end sigmoid colostomy. Surgical histopathology proved it to be sacral dedifferentiated chordoma and rectal invasive adenocarcinoma. Overall, the patient recovered well postoperatively, was discharged home with functional stoma and on permanent Foley catheter use.

**Conclusion:**

To the best of our knowledge, this is the only reported case of such a presentation, and sheds light on the approach and management. We hope that reporting such a case will add value to the medical literature.

**Supplementary Information:**

The online version contains supplementary material available at https://doi.org/10.1186/s13256-021-02728-2.

## Background

Chordoma is a low-grade, slow-growing tumor which originates from the notochord, the basis for the axial skeleton [1]. The incidence rate for chordomas is 0.1/100,000 per year. In addition, they involve the sacrococcygeal region in 50–60% of cases and account for over 40% of all sacral tumors [2]. It is quite rare to find a primary bone cancer in association with colorectal cancer [3]. Magnetic resonance imaging (MRI) is considered the best modality for diagnosing sacral chordoma [2]. Total surgical excision with negative margins is the main effective method as curative treatment [2–4].

## Case presentation

**Fig. 1:**
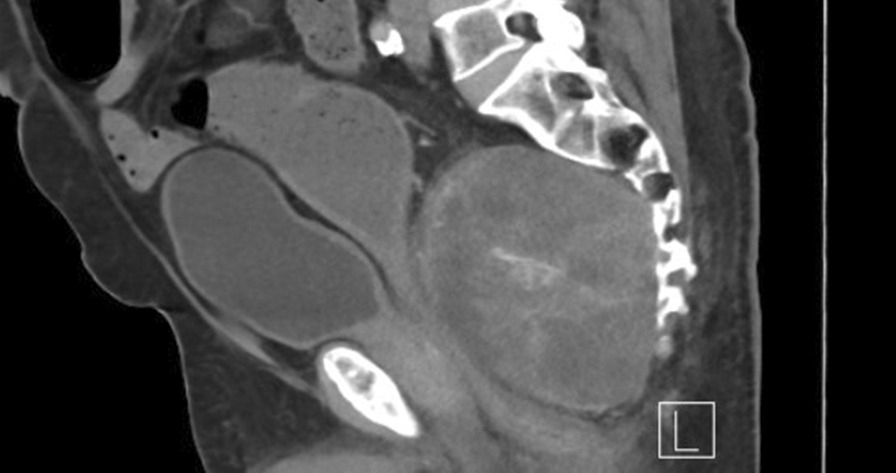
Lumbar computed tomography indicated a large midline, destructive, osseous, sacrococcygeal mass with a large presacral mass with soft tissue components and linear calcification measuring 12.5 × 10.4 × 10.6 cm

**Fig. 2:**
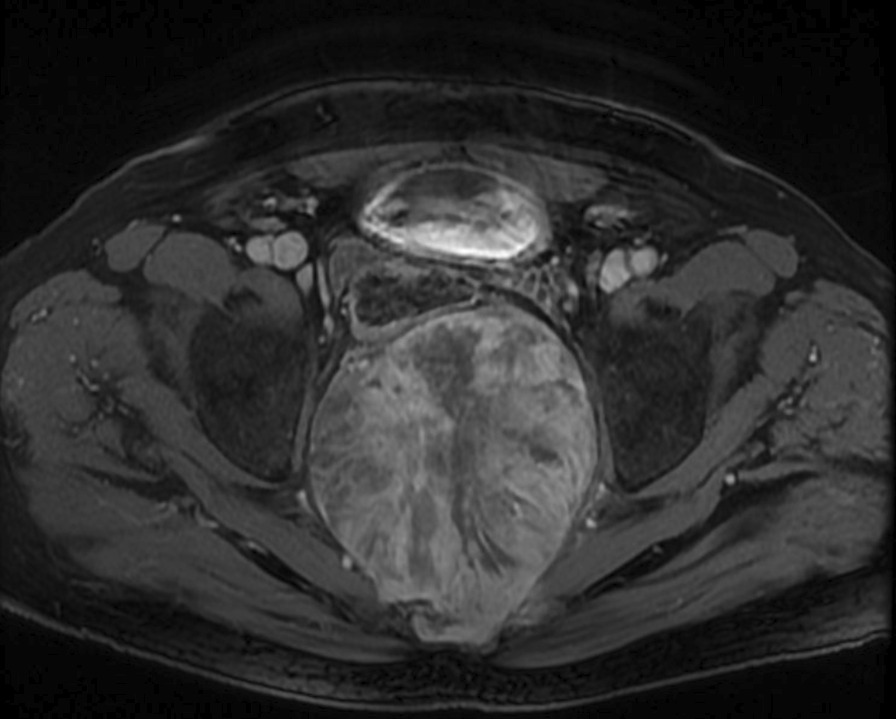
Magnetic resonance imaging of the pelvis showed a large midline, well-defined, oval shaped lesion replacing the sacrococcygeal portion of the spine, with extension to the presacral region

Our patient was a 74-year-old white middle eastern man, with known hypertension under treatment, who presented with complaints of back pain for the past 12 months which was progressive and localized at the lower back. The pain improved after receiving analgesia and was associated with episodes of urinary incontinence. The patient’s family history for cancers was unremarkable. He underwent investigations at a local hospital; the computed tomography (CT) scan showed a sacrococcygeal mass suggestive of chordoma. Therefore, the patient was referred to our center for further evaluation and management. He was seen in January 2020 in the outpatient orthopedic clinic at our hospital. The examination revealed localized tenderness at the coccygeal bone. Neurological tests (motor, sensory, and reflexes) were all normal. Blood test results were within normal limits. A lumbar CT indicated a large midline, destructive, osseous, sacrococcygeal mass with a large presacral mass with soft tissue components and linear calcification measuring 12.5 × 10.4 × 10.6 cm (Fig. 1). MRI of the pelvis showed a large midline, well-defined, oval-shaped lesion replacing the sacrococcygeal portion of the spine, with extension to the presacral region (Fig. 2). CT-guided Tru-Cut biopsy revealed features suggestive of dedifferentiated chordoma. The patient did not undergo colonoscopy, as he did not have rectal bleeding or changes in bowel habit, and the size of the mass was too large for colonoscopy to be performed or pass through. Moreover, neither MRI nor CT raised the suspicion of a rectal mass.

We decided to treat the tumor using a multidisciplinary approach that included a urology, colorectal, plastic, and orthopedic surgical team. First, the patient underwent cystoscopy and bilateral ureteral stenting. Afterwards, with the patient in a supine position, laparotomy was initiated, and the unilateral left pedicle rectus abdominis myocutaneous flap was harvested and saved to reconstruct the back defect after sacrectomy by the plastic surgery team. Subsequently, midline laparotomy incision was performed from the suprapubic site to 4 cm above the umbilicus. A 3M wound protector and Bookwalter wound retractors were applied. Dissection started laterally to medially until the sigmoid and superior rectal arteries from the inferior mesenteric artery were identified. Next, transection of the sigmoid parallel to the pedicle was performed using an NTLC stapler. Dissection of the rectum was then initiated from right to left until we reached below the sacral promontory to permit the sacrectomy procedure. Posterior rectal mobilization showed that we could not separate the rectum from the sacral mass, as it was very adherent and fixed. Therefore, to avoid any further disruption to the blood supply of the rectum, we proceeded with an end stoma. After that, the patient was moved to a prone position with the hips raised. A midline incision over the mass was made. An attempt made to dissect the tumor and free it from the surrounding structures and its attachment to the sacrum was successful. However, it was fixed to the sacrum and rectum. Therefore, sacrectomy was performed and the tumor with its attachment to the rectum was removed in one specimen. Next, L3, L4 and L5 pelvic fixation was done to provide further stabilization. The rectus abdominis flap was tunneled and delivered into the sacral defect. The flap had good color and blood supply. Therefore, it was fixed to the pelvic floor muscles using 2-0 Vicryl in a figure-of-eight fashion to approximate the defect. We then placed Surgimed mesh over the flap which was sutured and placed over the pelvic floor muscles using another 2-0 Vicryl in a horizontal mattress fashion. Lastly, minimal mobilization and release of the gluteus maximus muscle was performed with extra length for skin closure to avoid tension. Two Jackson-Pratt drains were emplaced. The patient tolerated the procedure and was transferred to the surgical intensive care unit.

**Fig. 3:**
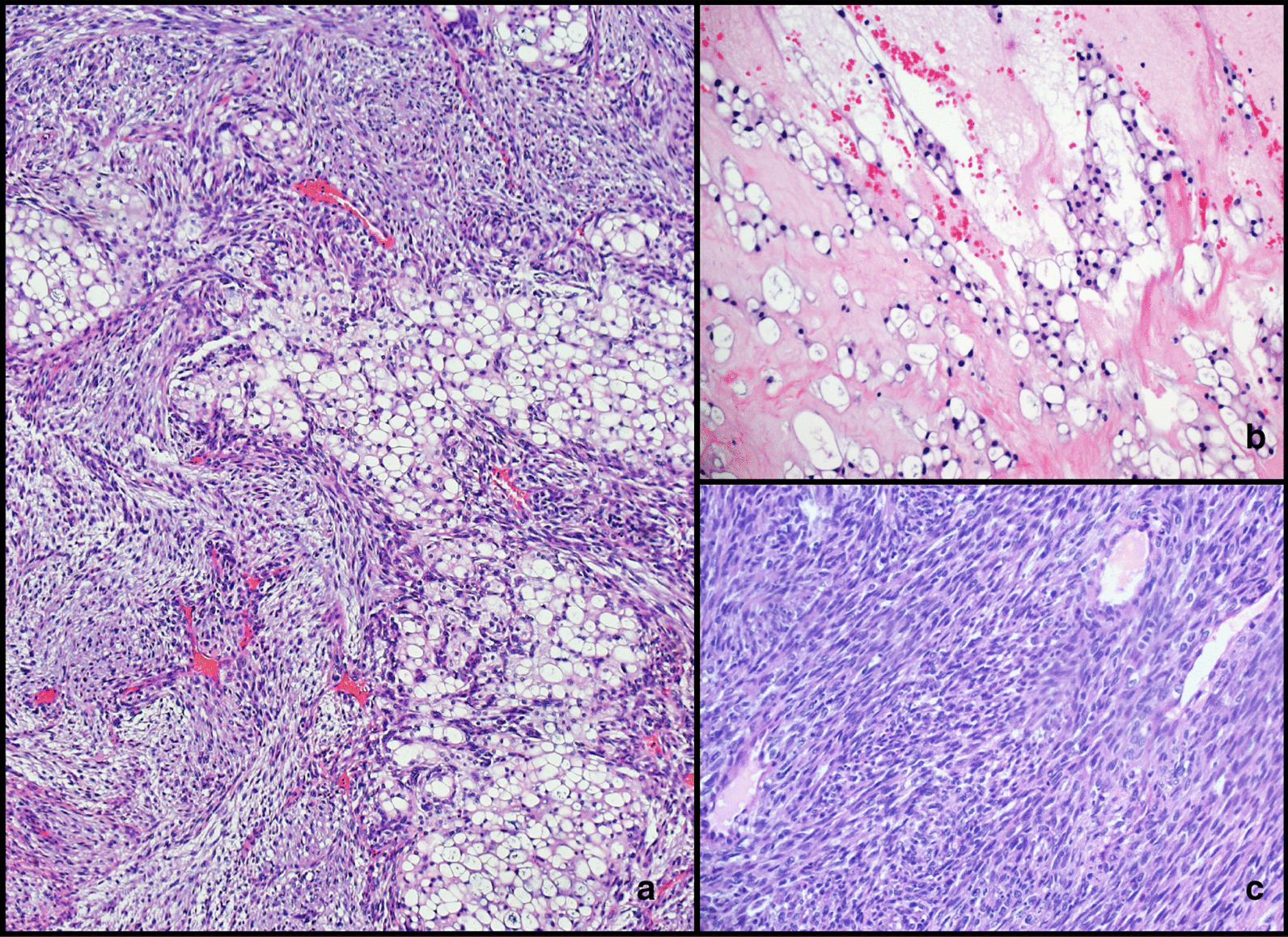
**a** High-power view of hematoxylin and eosin stained section showing epithelioid cells with pale eosinophilic and clear vacuolated cytoplasm consistent with chordoma. **b** High-power view of hematoxylin and eosin stained section showing highly atypical sarcomatoid spindle cell proliferation consistent with dedifferentiated area in chordoma. **c** Low-power view of hematoxylin and eosin stained section showing areas composed of cells with clear cytoplasm intermingled with cellular areas with spindle cell morphology

**Fig. 4:**
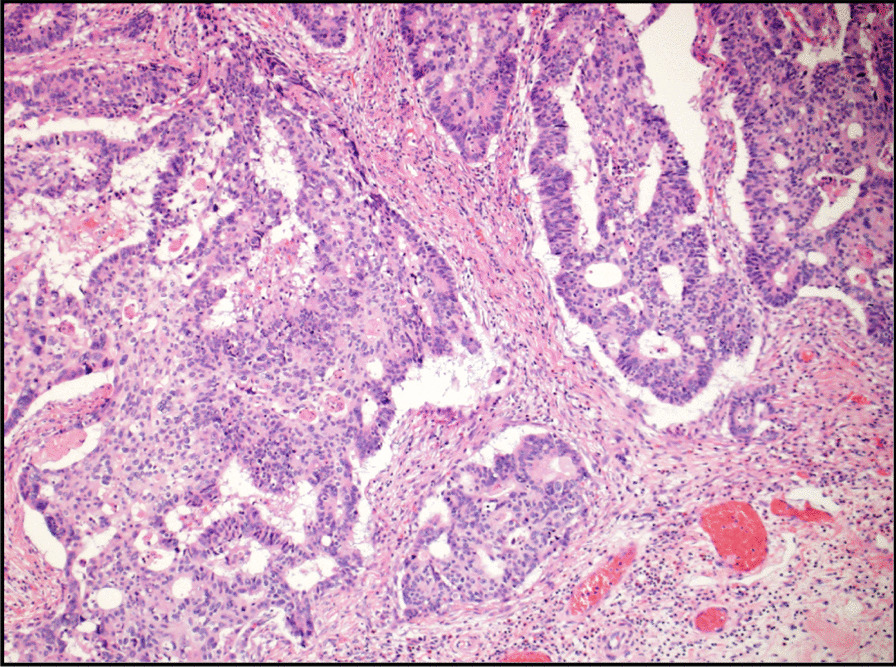
Microscopic view of hematoxylin and eosin stained section showing atypical glandular proliferation with cribriform pattern in a desmoplastic stroma consistent with invasive adenocarcinoma

Final histopathology of the resected specimen showed two different neoplasms: first, a sacral tumor consistent with dedifferentiated chordoma. Microscopically, the tumor was composed of cells with pale eosinophilic and clear vacuolated cytoplasm intermingled with high-grade sarcomatoid areas with spindle cell morphology (Fig. 3). The immunohistochemistry revealed positive staining in the pale eosinophilic/clear cells with epithelial membrane antigen (EMA), cytokeratin AE1/AE3 and S100 protein (focal). The second tumor was in the rectum, and microscopic examination revealed moderately differentiated adenocarcinoma invading the muscularis propria with no vascular or perineural invasion (Fig. 4). The surgical resection margins were free of tumor, and 16 lymph nodes were negative for metastasis; final staging was T2N0M0.

During the postoperative period, the patient was kept on a Foley catheter and prophylactic enoxaparin 40 mg. However, abdominal drains were slightly turbid in color and the patient had mild leukocytosis of 11.0 × 10^9^. Consequently, CT of the abdomen and pelvis were ordered to rule out intra-abdominal abscess, which showed a surgical site collection measuring 5.5 × 2.2 × 4.8 cm. The patient underwent ultrasound-guided drainage and the fluid was sent for culture. Microbiologically, the specimen revealed extended-spectrum beta-lactamase (ESBL) *Escherichia coli*; hence, the patient was treated with the appropriate intravenous antibiotic for an entire course of 14 days. In addition, upper cuts of the abdominal CT showed incidental findings of a left posterior segmental artery filling defect representative of pulmonary embolism despite the patient’s stable and asymptomatic status. Therefore, the anticoagulation team advised switching to rivaroxaban 20 mg daily. The patient was subsequently discharged home in good clinical condition, with functional stoma, all drains removed, healthy surgical wound, and on permanent Foley catheter, as his urinary incontinence did not improve. On follow-up 4 months after surgery, he remained stable, and his lab tests and investigations were unremarkable.

## Discussion and conclusions

Chordoma is a rare malignant bone tumor that emerges from notochordal remnants and occurs in the axial skeleton, with a tendency for the sacrum, base of the skull and mobile spine [4]. Chordomas occur between the fourth and seventh decades of life, and peak in the fifth decade, with a male-to-female ratio of 2–3:1.5. Clinical signs and symptoms depend on the location, the size of the tumor and the extent of neural invasion. Symptoms of sacral chordomas vary from pain, numbness and constipation, to weakness and incontinence [5]. On the other hand, retrorectal tumors are a rare disease with a variety of pathologies occurring in a potential space between the rectum and sacrum [4].

Grossly, sacrococcygeal chordomas are mostly well demarcated by a pseudocapsule and involve the bone and surrounding tissues. The surface of the tumor is soft, gelatinous, mucoid and hemorrhagic. Chordomas are classified as classical, chondroid and dedifferentiated types. The most common is the classical type, and in terms of prognosis, the worst is the dedifferentiated type. Histologically, it consists of chords of tumor cells and lobules with a mucoid matrix and extensive fibrous tissue in between [5].

Chordoma is best seen on T2-weighted MRI. MRI of the entire spine should be performed to check for any metastasis. CT is recommended in addition to MRI if the diagnosis of chordoma is vague. Furthermore, CT scans of the chest, abdomen and pelvis are recommended to check for any tumor spread [6].

Typically, such major surgery involving a large retrorectal tumor with locoregional invasion is expected to carry a risk of a wide range of neurological defects, for instance, incontinence and sexual dysfunction due to extensive sacral nerve root excision [1]. Therefore, we anticipated that long-term Foley catheter use might be necessary for our patient after the operation. There is no clear surgical approach or technique mentioned in the literature in such a major case. Therefore, we decided to proceed with a multidisciplinary approach consisting of urology, colorectal, plastic and orthopedic surgery. The urology team started by cystoscopy and bilateral ureteral stenting insertion; the orthopedic team was able to excise the entire tumor in an en bloc sacrectomy, and lumbopelvic fixation. The colorectal surgical team decided intraoperatively to proceed with end sigmoid colostomy, with a 6 cm closed distal rectal stump, to avoid morbidity and mortality risk of abdominoperineal resection. Anterior resection was also not an option because the patient underwent sacral nerve dissection. Therefore, to avoid fecal incontinence, the plastic surgery team secured a viable rectus abdominis flap for wound defect reconstruction. Together, this led to the success of the procedure and overall patient outcome.

Chemotherapy was not necessary for our patient for the rectal cancer because we had negative margins, and 16 lymph nodes were negative for metastasis (T2N0M0). However, the effects of adjuvant therapies such as chemotherapy and radiation therapy for chordoma are debatable and not as effective as surgery. There have been some indications for radiotherapy mentioned in the literature, as it may be helpful in prolonging the disease-free interval. Such indications include inoperable masses, no free surgical margins or incomplete excision of the mass. Also, radiation can be used after resection of the primary tumor or if recurrence is visible on follow-up imaging [4, 7].

Prognosis depends on the surgical resection of the tumor and postoperative treatment. However, the possibility of metastasis cannot be ruled out, and local recurrence usually has a devastating outcome. The five-year survival rate is 51%, and the 10-year survival rate is 35% [2].

In conclusion, we experienced a unique and rare case of synchronous sacral chordoma associated with rectal adenocarcinoma. To the best of our knowledge, this is the only case of such a presentation in the medical literature. Approaching such a major case through a multidisciplinary team approach was a crucial step in its successful outcome, which was reflected in the patient’s overall recovery.

## Supplementary Information

**Additional file 1:** Surgical specimen side view. courtesy King Faisal Specialist Hospital and Research Center.

**Additional file 2:** Surgical specimen top view. courtesy King Faisal Specialist Hospital and Research Center.

## Data Availability

The data sets used and/or analysed during the current study are available from the corresponding author on reasonable request.

## Abbreviations

MRI: Magnetic resonance imaging
CT: Computed tomography
ESBL: Extended-spectrum beta-lactamase
