# Supplementary figures and images for: Sacral chordoma with incidental rectal adenocarcinoma: a case report
Source: J Med Case Rep. 2021 Apr 12;15:195. doi: 10.1186/s13256-021-02728-2 (PMC8040199; doi:10.1186/s13256-021-02728-2)

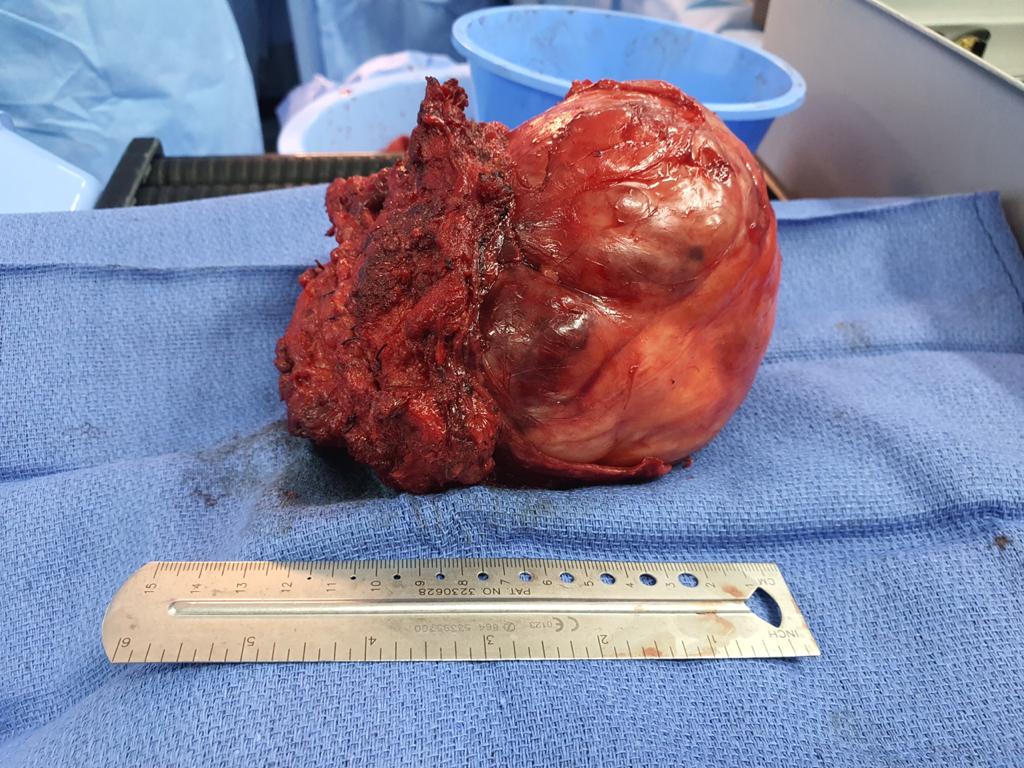

Supplement: Supplementary file 1 — Additional file 1: Surgical specimen side view. courtesy King Faisal Specialist Hospital and Research Center. [file 13256_2021_2728_MOESM1_ESM.jpg]

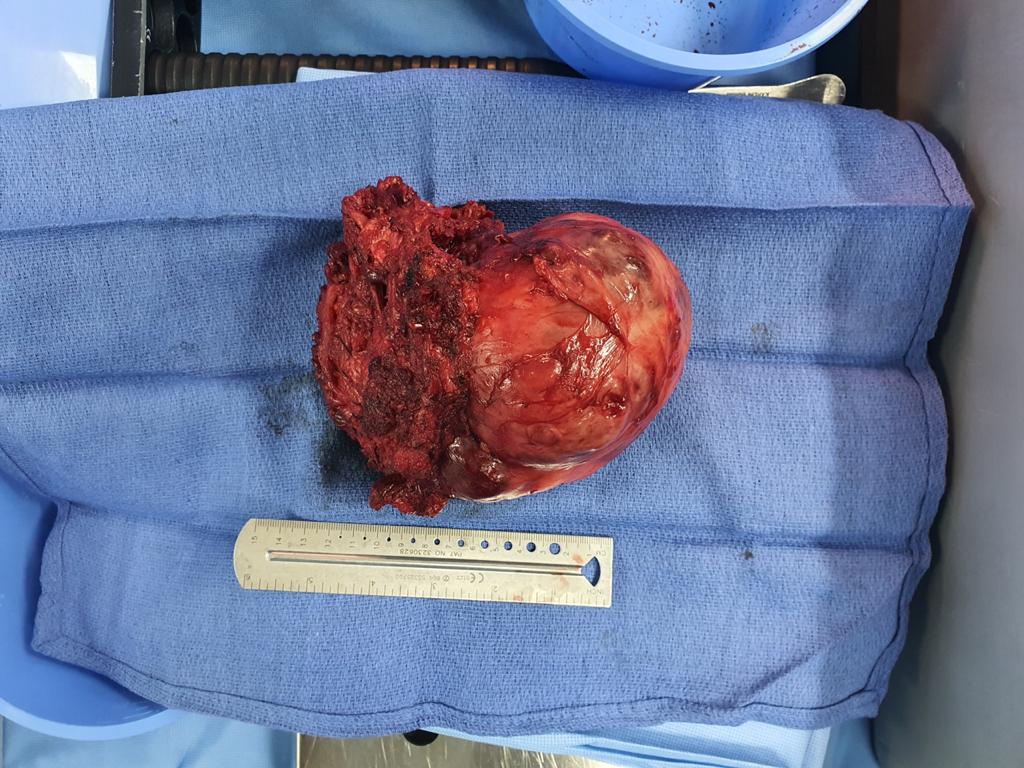

Supplement: Supplementary file 2 — Additional file 2: Surgical specimen top view. courtesy King Faisal Specialist Hospital and Research Center. [file 13256_2021_2728_MOESM2_ESM.jpg]
